# The perception of nursing leaders towards communication and relationship management competencies in using digital platforms during COVID‐19 in Qatar: A cross‐sectional study

**DOI:** 10.1111/jonm.13722

**Published:** 2022-07-03

**Authors:** Laith F. Daradkeh, Ralph C. Villar, Abdulqadir J. Nashwan

**Affiliations:** ^1^ Department of Nursing, Hazm Mebaireek General Hospital (HMGH) Hamad Medical Corporation (HMC) Doha Qatar

**Keywords:** nursing, leadership, COVID‐19, management, communication

## Abstract

**Aim:**

To evaluate nursing leaders' perception of communication and relationship management competencies while using digital platforms during the COVID‐19 pandemic.

**Background:**

Nursing leaders can achieve effective leadership by mastering these competencies leading to an overall improvement in the quality of nursing care. The COVID‐19 pandemic has brought numerous challenges in communication, and digital platforms have been widely used in healthcare settings to mitigate contagion.

**Design:**

Cross‐sectional.

**Methods:**

The study was conducted between February and March 2021. A survey was adopted from the American Organization of Nurse Executives (AONE) and was sent to nursing leaders in Qatar through email.

**Results:**

A total of 250 nurse leaders were invited to participate, but only 116 responded (RR 46.4%). The male participants represented a more significant proportion of 64.10%. Influencing behaviour, relationship management and effective communication had the lowest scores, which indicates low competency.

**Conclusions:**

Despite obtaining satisfying scores, nursing leaders in Qatar should strive for professional development and knowledge acquisition to improve their communications and relationship management competencies.

**Implications for Nursing Management:**

Healthcare organizations must understand that nursing leaders should strive for professional development and knowledge acquisition to improve their communication and management.

## 1 BACKGROUND

Nowadays, nursing leaders must have advanced management talent to correspond to the continuously increasing demands and complexity in healthcare provision (Cheryl Lacasse, 2013; Uhl‐Bien et al., 2020). Nursing leaders were expected to show measurable outcomes and efficiency to facilitate effective practice with evidence‐based management. Most academic studies and nursing leadership programmes emphasize on competencies related to working environment effectiveness. The shift to evidence‐based management has led to numerous efforts to define appropriate competencies for nursing leaders (Bianchi et al., 2018; Stefl, 2008). For example, communication and relationship management competency relates to how the leaders understand the individuals they work with and how they use their knowledge effectively in developing high‐performance workplace relationships, in addition to how they utilize digital platforms to manage their working environment (Garman & Johnson, 2006; Temelkova, 2018). Overlooking the leadership skills for nurse executives and managers can have negative consequences, and self‐reported competency can yield positive results (Flatekval & Corbo, 2019).

The American Organization of Nurse Executives (AONE), which was a member of the Healthcare Leadership Alliance (HLA), defined eight main domains correlated with this competency (Garman & Johnson, 2006). Most of these domains concentrate on communication and relationship management at the organizational level (e.g. understanding the corporation's structure and relationships, public relations and communicating the corporation's mission and vision). Other domains concentrate on the departmental level (e.g. promoting alternative conflict resolution, practising and evaluating shared decision making and building, engaging in and leading teams) (Reid Ponte, 2004). However, mastering these competencies was only an entry ticket for leadership effectiveness, and the competency should be continuously utilized within the daily practice as part of continuous professional development. Over time, with constant utilization, this competency can significantly improve the effectiveness of nursing leadership and thus the quality of nursing care delivered to service users.

The COVID‐19 pandemic has created an urgent need for coordinated mechanisms to respond to outbreaks across health sectors, and digital health solutions have been identified as promising approaches to meet this challenge (Allobaney et al., 2020; Nashwan, Abujaber, Mohamed, et al., 2021; Nashwan, Abujaber, Villar, et al., 2021; Nashwan, Villar, Al‐Qudimat, et al., 2021; Villar et al., 2021). Digital platforms are technologies used to boost interactions among people, offering more effective ways of transferring information (Caputo et al., 2018). The use of digital platforms during the pandemic has been a critical ally in addressing issues. Telemedicine was used to reduce the risk of infection as a successful healthcare model in both emergency and primary care (Fagherazzi et al., 2020). Official communication plans should promote accessible and varied channels to inform people of the epidemic, avoid rumours and reduce threats to public health. Social media platforms such as Microsoft Teams™ and Google Trends were beneficial for modelling trends in the epidemic and monitoring the evolution of patient symptoms or general reactions and outcomes of the epidemic over time. Numerous studies agree that the pandemic exacerbated and accelerated digital globalization, especially in healthcare (Alexopoulos et al., 2020; Liang, 2020; Parker et al., 2021; Popescu et al., 2021).

The purpose of this study was to identify the perceived level of nursing leaders' communication and relationship management competencies when using digital platforms. The objectives of the study were (1) to evaluate nursing leaders' perception towards communication and relationship management competencies while using digital platforms during the COVID‐19 pandemic and (2) to compare different professional levels (executive director of nursing, assistant executive director of nursing, director of nursing and head nurses of various units) communication and management competencies. Specifically, the study wants to answer the following questions: What is the level of perception towards communications and relationship management in using digital platforms during COVID‐19 among nursing leaders in Hamad Medical Corporation (HMC), and is there a difference in the level of perception towards communications and relationship management in using digital platforms during COVID‐19 among nursing leaders in Hamad Medical Corporation? As of the researcher's knowledge, this was the first study on nursing leaders' perception of their communication and relationship management using digital platforms during the pandemic.

## 2 MATERIALS AND METHODS

This study was conducted in different hospitals under Hamad Medical Corporation in Qatar, the largest healthcare provider in the country. A cross‐sectional, descriptive study design was used to answer the research questions from February to March 2021. The survey was distributed to a purposive sample of nursing leaders in Hamad Medical Corporation (using their organizational contact information), including executive directors, assistant executive directors, directors of nursing and head nurses at various units/departments. The expected response rate according to Qualtrics Sample Size Calculator for 250 population (confidence level 95%) from executive director of nursing, assistant executive director of nursing, director of nursing and head nurses of various units; from the eligible participants, 116 responded with a 46.4% response rate.

### 2.1 Data collection

An official email was sent from the researcher through the HMC research department to all targeted participants, explaining the nature and scope of the study and the voluntary nature of participation, including the right to withdraw at any time and the right to anonymity. Completion of the questionnaire was considered as approval of participation. Participants were informed that they had 2 weeks to fill in the questionnaire. Reminders were sent by email to complete the survey to improve the response rate. A 5‐item Likert‐type questionnaire adopted from the AONE (2005) questionnaire to evaluate communication and relationship management competencies. Benner's (1982) beginner to expert theory provides the foundation for this instrument. For the professional nurse, Benner recognized five levels of development: novice, advanced beginning, competent, proficient and expert. The survey has two sections: (1) demographics such as gender, years of experience in general and particularly in HMC, as well as the current managerial role. The second part consists of eight competencies including (1) effective communication (three items), (2) relationship management (seven items), (3) influence of behaviours (three items), (4) ability to work with diversity (six items), (5) shared decision‐making (three items), (6) community involvement (three items), (7) medical staff relationships (eight items) and (8) academic relationships (six items).

### 2.2 Ethical approval

The study was conducted in full conformance with principles of the ‘Declaration of Helsinki’, Good Clinical Practice (GCP), and within the laws and regulations of MoPH in Qatar. Implied consent was obtained from the participants, and the researcher assured voluntary participation for the subjects. Also, the questionnaire was disseminated without names or corporation numbers to guarantee participant anonymity and data confidentiality. Original authors provided the researcher with permission to use the scales. The researcher adhered to the ethical codes and regulations of the University of Essex and got approval from the IRB of Hamad Medical Corporation (MRC‐01‐21‐090).

## 3 STATISTICAL ANALYSIS

The IBM Corp. Released 2017. IBM SPSS Statistics for Windows, Version 25.0. Armonk, NY: IBM Corp., was used to analyze the data. Descriptive statistics, including percentages, frequencies, means and standard deviations, were used to describe the sample and answer the research questions about the perception of communication and relationship management competencies. In addition, ANOVA and Wilcoxon‐rank sum tests were done to compare the status of communications and relationship management competencies.

Assumptions were checked and insured before using the inferential statistics. The researcher reviewed the outliers and the missing values and dealt with them appropriately. Then, the researcher ran the analysis. The data were considered significant when *P* value < .05.

## 4 RESULTS

The majority of participants were male, consisting 63.8% of the sample size, whereas women were 36.2%. Nursing leaders were represented in all departments where 60.4% were head nurses, 28.4% were directors of nursing and 11.2% were executive directors and assistant executive directors of nursing. Years of experience ranged from 0 to above 10 years. The majority of the participants had over 10 years of experience (53.5%) (Table 1).

**TABLE 1:** Participants socio‐demographics and work‐related factors (*n* = 116)

Variables		Frequency	Percentage
Gender	Male	74	63.8
	Female	42	36.2
Current position in HMC	Executive director of nursing	13	11.2
	Assistant executive director of nursing		
	Director of nursing	33	28.4
	Head nurse	70	60.4
Experience in current position	0–5 years	23	19.8
	5–10 years	31	26.7
	Above 10 years	62	53.5

The results of the study showed that all the items revealed a sufficient level of internal consistency with Cronbach alpha (α ˃ .987) (Table 2).

**TABLE 2:** Reliability test

	Scale mean if item deleted	Scale variance if item deleted	Corrected item‐total correlation	Cronbach's alpha if item deleted
Effective communication	122.560	13301.348	.996	.981
Relationship management	122.940	13529.473	.993	.981
Influencing behaviours	122.760	13529.068	.997	.981
Shared decision making	123.720	13465.312	.850	.991
Community involvement	123.640	13770.793	.865	.989
Medical staff relationship	123.340	13217.923	.975	.982
Academic relationship	123.240	13568.908	.982	.982
Cronbach's alpha	Cronbach's alpha based on standardized items			*N* of items
.986	.987			7

*Note*: The results of the study showed that all the items revealed consistency.

This study was conducted in different hospitals under HMC in Qatar, the largest healthcare provider in the country.

### 4.1 Descriptive statistics

A participant can gain a maximum mean score of 52.6, which is the highest level of perception on the communication and relationship skills, and a minimum of 0, which means the participant has the poorest communication and relationship skills. The participants of this study had the highest mean score on effective communication (21.14), followed by influencing behaviours (20.94), relationship management (20.76), academic relationship (20.46), medical staff relationship (20.36), community involvement (20.06) and the lowest mean score on shared decision making (19.98). The items of digital competency, effective communication, relationship management and influencing behaviour obtained the lowest scores, with 44.0, 42.0 and 43.0, indicating they have a low level of competency (Table 3).

**TABLE 3:** Nursing leaders' communication and relationship management competency scores

	*N*	Minimum	Maximum	Mean	Std. deviation
Effective communication	5	.0	44.0	21.140	20.0486
Relationship management	5	3.0	42.0	20.760	19.1230
Influencing behaviours	5	3.0	43.0	20.940	19.0491
Shared decision making	5	.0	52.6	19.980	22.0819
Community involvement	5	2.0	52.0	20.060	20.3264
Medical staff relationship	5	.8	49.8	20.360	20.7963
Academic relationship	5	1.5	44.5	20.460	19.1346
Valid *N* (listwise)	5				

*Notes*: The participants of this study had the highest mean score on effective communication, followed by influencing behaviours, relationship management, academic relationship, medical staff relationship and community involvement, and the lowest mean score on shared decision making. The items of digital competency, effective communication, relationship management and influencing behaviour obtained the lowest scores, indicating they have a low level of competency.

### 4.2 Differences in nurses' leaders perception of communication and relationship management competency in using digital platforms

The participants rated the overall communications and relationship management competencies and proficiency level related to digital platform utilization by nurse leaders. The items have an *F* value close to 1.0, indicating that the null hypothesis is true. The significance suggests that even though there is a difference in response on effective communication proficiency of nurse leaders, the difference is statistically significant because it is less than .05. That implies that it would occur 41.1% in influencing behaviour, 40.9% in relationship management, 40.4% in shared decision making, 40.1% in community involvement, 23.4% in medical staff relationship and 40.3% in an academic relationship (Table 4).

**TABLE 4:** Differences in nurse leaders' perception of communication and relationship management competency

		Sum of squares	df	Mean square	*F*	Sig.
Influencing behaviours	Between groups	1451.472	4	362.868	.677	.411
	Within groups	.000	0			
	Total	1451.472	4			
Relationship management	Between groups	1462.752	4	365.688	.681	.409
	Within groups	.000	0			
	Total	1462.752	4			
Shared decision making	Between groups	1950.448	4	487.612	.887	.404
	Within groups	.000	0			
	Total	1950.448	4			
Community involvement	Between groups	1652.652	4	413.163	.729	.401
	Within groups	.000	0			
	Total	1652.652	4			
Medical staff relationship	Between groups	1729.952	4	432.488	.912	.234
	Within groups	.000	0			
	Total	1729.952	4			
Academic relationship	Between groups	1464.532	4	366.133	.793.	.403
	Within groups	.000	0			
	Total	1464.532	4			

*Notes*: Statistical significance determined by *P* < .05. The items have an *F* value close to 1.0, indicating that the null hypothesis is true.

Abbreviation: df, degree of freedom.

## 5 DISCUSSION

This study identified the perceived level of nursing leaders' communication and relationship management competencies when using digital platforms. This result offered practical insight for evaluating expertise in crucial communication and relationship management competencies. Lucas et al. (2018) argue that the competency assessment tools may be utilized in various ways to detect strengths and improvement areas. This result can enhance the following areas: (1) Group or team development as the tool can help link individual goals of a team to the organizational objectives, resulting in corporate values, goals and objectives. (2) Organizational and self‐assessment provide valuable information of strengths and weaknesses in communication and relationship management competencies to inform a professional improvement strategy. (3) Professional and academic development programmes can help uncover skills, knowledge and specific competencies that programmes should focus on when offering professional development programmes. This study revealed that there was no difference in the nursing leaders' perception of communication and management competencies in using digital platforms during COVID‐19 in Qatar. Despite being competent in all aspects of communication and relationship management, it was essential to note that low levels were recorded in digital competency, effective communication, relationship management and influencing behaviour. Furthermore, in the proficiency level subscale, participants scored the highest mean on effective communication, and the lowest mean in shared decision making. Influencing behaviour scored the lowest standard deviation, and shared decision making had the highest standard deviation indicating minimal dispersion in the data. The digital platforms that enhance professional networking were only available and cater to individuals within these nursing professions (Krawczyk‐Sołtys, 2017). In addition, discussions and clinical topics in such sites often address diverse subjects, including biostatistics, ethics, practice management, politics and career strategies (Chen, 2018). The results from this study indicated that digital platforms provide a supportive environment for nursing leaders. Digital platforms also enhance crowdsourcing and involve harnessing society's skills and knowledge to gather opinions and information or solve problems (Shum et al., 2018). According to Hernandez et al. (2018), social media connects nurse leaders in developing countries with those from more medically advanced nations. Digital platforms connect a broader nursing audience and magnify content and critical themes (Hernandez et al., 2018). From the responses in the survey, nurse leaders who use digital platforms, the assistance and information received were helpful. In the descriptive table, shared decision making and community involvement had the highest maximum values of 52.6 and 52.0.

According to Lucas et al. (2018), nurse leaders can ask questions on Twitter or stream surgical procedures through the Internet (Lucas et al., 2018). Therefore, digital platforms offer a new communication channel for nurse leaders to network, exchange and share medical information in ways and at a pace that was never possible before.

The improved communication provided by digital platforms plays a critical role in improving clinical education. Hernandez et al. (2018) argue that the high utilization rate of digital platforms by individuals between 18 and 29 years fostered the adaptation of clinical curricula to reflect incoming students' culture and changing habits (Hernandez et al., 2018). This study's results indicate that the *F* value of the academic relationship is .793, indicating the null hypothesis is true. That implies that nurse leaders actively use digital platforms to improve students' understanding of ethics, communication and professionalism.

The focus of the benefits of digital platforms in enhancing nursing leaders' work within the society came across strongly because of the need to provide integrated social and healthcare services in Qatar and the international emphasis to improve primary care to support patient self‐management at home (Handtke et al., 2019). The nursing workforce needs to have a high level of generalized information and coordination, excellent communication and leadership skills, which will ensure they can navigate the complex online environment and effectively deliver healthcare services to the community. Furthermore, the advanced practice roles catering to the needs of patients will be critical for the aging population as it is now a global trend (Lucas et al., 2018). Therefore, there is a need to research the specialized healthcare services that the patients require, develop and implement them in nursing practice, education, the level of demand and policies governing the integration agenda, ensuring nurses were adequately equipped for the future.

COVID‐19 increased the need to deliver care remotely through networked care delivery and mobile consultations to limit the need for direct contact. Mast et al. (2018) argued that because healthcare providers will not physically examine the patients, clear guidance and good support were critical to ensure effective treatment and diagnosis at a distance (Mast et al., 2018). The findings of the study showed that digital platforms allow nursing leaders to provide ongoing guidance and support. In addition, the digital platform creates a need for nursing leaders to provide knowledge to patients to ensure they can evaluate and interpret the information from monitoring mechanisms to empower their decision‐making process.

Nursing leaders should ensure transparent and clear communication between the user and the professional. The clarity, listening skills and voice intonation of virtual communication information are essential as face‐to‐face support and prompts are absent. For example, healthcare professionals may communicate through video or audio connections provided on digital platforms (Collins et al., 2017). This study's findings indicated that specific competencies needed for remote healthcare service provision include providing consultations to the community, being an influential member of professional organizations, representing organizations of non‐health within the community.

Chen (2018) argued that nurse leaders may be required to engage other healthcare providers when making healthcare‐related decisions and should ensure that the patients' health information is shared responsibly. The study highlighted confidentiality concerns and argued that digital platforms should enhance the responsible sharing of information across and between organizations. However, nursing leaders should ensure regular communication to improve teamwork with other healthcare shareholders such as healthcare organizations, communities, caregivers, nurses, physicians and voluntary groups. In addition, nursing leaders must be aware of the implications of digital platforms across a broader integrated spectrum of healthcare services.

There is a risk of breaching patients' confidentiality and privacy when nurses share patient information during interactions on digital platforms (Krawczyk‐Sołtys, 2017). Nurse leaders should be aware of the specific ways that digital platforms compromise patients' confidentiality and privacy and how patients' right to know how the information is viewed and shared. Although most healthcare organizations have standards for data sharing, digital platforms alter how healthcare providers view and share information. It results in unique patient data requirements and how they can be kept secure from others using online tools and mobile devices (Shum et al., 2018). Because digital platforms draw nursing leaders from various organizations, they must monitor information access (Handtke et al., 2019). They need to enhance effective information sharing across nurse leaders while considering legislation rules and national privacy laws.

## 6 STRENGTHS AND LIMITATIONS

This research was generated within a short period; therefore, it might affect the generalization. In addition, the study had a small sample population, and the time of introduction of the survey may lead to information bias. The sample consisted of 116 participants, representing a 46.4% response rate. Similar research should be conducted with a larger sample size to ascertain the results.

## 7 CONCLUSIONS

Qatar's nursing leaders should capitalize on communication and relationship management in using digital platforms because of their benefits and capability to generate critical discussions, especially during pandemics. Despite obtaining satisfying scores, nursing leaders in Qatar should strive for professional development and knowledge acquisition to improve their communications and relationship management competencies.

## 8 IMPLICATIONS FOR NURSING MANAGEMENT

Increased use of digital platforms in the healthcare setting creates a need for experts to research them to identify the bottlenecks associated with them and discover ways to successfully implement digital platforms in healthcare settings. Nursing leaders gain from including digital platforms in their engagement strategies, particularly during the COVID‐19 pandemic, and should consider the following when using digital platforms to enhance healthcare service provision. First, they should consider the social media policies issued by a professional association or within the healthcare organization. Nurse leaders should also assess how analytics in digital platforms were driven and designed. There were some standard metrics, but the use and definition of platforms vary; therefore, they can be misleading. For instance, in Twitter, the term impressions refer to tweets posted to a tweeter stream. The impressions exclude social media information streamed to third‐party applications such as Hootsuite. Therefore, the analytics reflect the size of the potential audience. A need for more nursing research will help understand the disadvantages and advantages of digital platform analytics, which will help educate nurse leaders and ensure nurses understand the drawbacks and benefits in interpreting virtual behaviours and online environments. In order to further understand the phenomena, a qualitative research methodology must be considered.

## CONFLICT OF INTEREST

The authors declare that they have no competing interests.

## ETHICS STATEMENT

The study was approved by the Medical Research Center (MRC) Institutional Review Board (IRB) at Hamad Medical Corporation (MRC‐01‐21‐090). Implied consent was obtained from the participants, and the researcher assured voluntary participation for the subjects. Also, the questionnaire was disseminated without names or corporation numbers to assure participant anonymity and data confidentiality.

## AUTHOR CONTRIBUTIONS

Conceptualization: LFD. Methodology: LFD, RCV and AJN. Formal analysis: LFD. Manuscript draft writing: LFD, RCV and AJN. Manuscript final editing: LFD, RCV and AJN.

## Data Availability

All data generated during this study are included in this published article.
